# Toll‐like receptor 7/8‐matured RNA‐transduced dendritic cells as post‐remission therapy in acute myeloid leukaemia: results of a phase I trial

**DOI:** 10.1002/cti2.1117

**Published:** 2020-03-03

**Authors:** Felix S Lichtenegger, Frauke M Schnorfeil, Maurine Rothe, Katrin Deiser, Torben Altmann, Veit L Bücklein, Thomas Köhnke, Christian Augsberger, Nikola P Konstandin, Karsten Spiekermann, Andreas Moosmann, Stephan Boehm, Melanie Boxberg, Mirjam HM Heemskerk, Dennis Goerlich, Georg Wittmann, Beate Wagner, Wolfgang Hiddemann, Dolores J Schendel, Gunnar Kvalheim, Iris Bigalke, Marion Subklewe

**Affiliations:** ^1^ Department of Medicine III University Hospital, LMU Munich Munich Germany; ^2^ Laboratory for Translational Cancer Immunology Gene Center LMU Munich Munich Germany; ^3^ German Cancer Consortium (DKTK) and German Cancer Research Center (DKFZ) Heidelberg Germany; ^4^ DZIF Research Group “Host Control of Viral Latency and Reactivation” (HOCOVLAR) Helmholtz Zentrum München Munich Germany; ^5^ Max von Pettenkofer Institute LMU Munich Munich Germany; ^6^ Institute of Pathology Technical University of Munich Munich Germany; ^7^ Department of Hematology Leiden University Medical Center Leiden The Netherlands; ^8^ Institute of Biostatistics and Clinical Research University of Muenster Muenster Germany; ^9^ Department of Transfusion Medicine, Cellular Therapeutics and Hemostaseology University Hospital LMU Munich Munich Germany; ^10^ Medigene AG Planegg Germany; ^11^ Department of Cellular Therapy The Norwegian Radium Hospital Oslo University Hospital Oslo Norway; ^12^ Present address: Roche Innovation Center Munich Penzberg Germany; ^13^ Present address: Medigene AG Planegg Germany; ^14^ Present address: BioNTech IMFS Idar‐Oberstein Germany

**Keywords:** acute myeloid leukaemia, cancer vaccines, clinical trials, dendritic cell vaccination, immunotherapy

## Abstract

**Objectives:**

Innovative post‐remission therapies are needed to eliminate residual AML cells. DC vaccination is a promising strategy to induce anti‐leukaemic immune responses.

**Methods:**

We conducted a first‐in‐human phase I study using TLR7/8‐matured DCs transfected with RNA encoding the two AML‐associated antigens WT1 and PRAME as well as CMVpp65. AML patients in CR at high risk of relapse were vaccinated 10× over 26 weeks.

**Results:**

Despite heavy pretreatment, DCs of sufficient number and quality were generated from a single leukapheresis in 11/12 cases, and 10 patients were vaccinated. Administration was safe and resulted in local inflammatory responses with dense T‐cell infiltration. In peripheral blood, increased antigen‐specific CD8^+^ T cells were seen for WT1 (2/10), PRAME (4/10) and CMVpp65 (9/10). For CMVpp65, increased CD4^+^ T cells were detected in 4/7 patients, and an antibody response was induced in 3/7 initially seronegative patients. Median OS was not reached after 1057 days; median RFS was 1084 days. A positive correlation was observed between clinical benefit and younger age as well as mounting of antigen‐specific immune responses.

**Conclusions:**

Administration of TLR7/8‐matured DCs to AML patients in CR at high risk of relapse was feasible and safe and resulted in induction of antigen‐specific immune responses. Clinical benefit appeared to occur more likely in patients <65 and in patients mounting an immune response. Our observations need to be validated in a larger patient cohort. We hypothesise that TLR7/8 DC vaccination strategies should be combined with hypomethylating agents or checkpoint inhibition to augment immune responses.

**Trial registration:**

The study was registered at https://clinicaltrials.gov on 17 October 2012 (NCT01734304) and at https://www.clinicaltrialsregister.eu (EudraCT‐Number 2010‐022446‐24) on 10 October 2013.

## Graphical abstract

Dendritic cell (DC) vaccination is a promising strategy to induce anti‐leukaemic immune responses. In this first‐in‐human phase I trial, TLR7/8‐matured DCs transfected with RNA encoding two leukaemia‐associated antigens (WT1 and PRAME) and CMVpp65 were used as post‐remission therapy for AML patients at high risk of relapse. DC generation was feasible, and administration was safe and resulted in local inflammatory responses and expanded antigen‐specific CD8^+^ and CD4^+^ T cells in peripheral blood; clinical benefit correlated with younger age and immune responders.

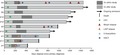

## Introduction

Despite improvements in outcome over the past decades, with 5‐year survival rates climbing from 6.2% in 1975–1977 to 28.1% in 2008–2014^1^ acute myeloid leukaemia (AML) still has a dismal prognosis.^2^ The major reason for the poor survival rate is the high risk of relapse after intensive induction therapy. The most successful strategy to reduce the relapse rate is allogeneic haematopoietic stem cell transplantation (allo‐HSCT).^3^ This potentially curative cellular immunotherapy is based on the graft‐versus‐leukaemia effect of allogeneic T cells. However, because of high morbidity and mortality of this therapy, there is a large group of AML patients without this therapeutic option. Alternative strategies for the activation of the immune system aiming at eradication of chemorefractory residual disease are therefore urgently sought after. Vaccines induce and enhance autologous T cells targeting intracellular leukaemia‐associated antigens (LAAs) and represent a promising strategy. Immunisation with LAA peptides has been studied in several clinical trials with moderate clinical success so far.^4^, ^5^ Optimisation of vaccination might be achieved by the use of DCs. As professional antigen‐presenting cells, they represent physiological candidates to induce strong and durable immune responses.^6^, ^7^ Several strategies have been applied including hybridomas of autologous DCs fused with leukaemic blasts from primary diagnosis as a vaccine in 17 AML patients in CR. Immunological responses were observed, and 71% of the patients were still in CR at a median follow‐up of almost 5 years.^8^ Results of two major studies using monocyte‐derived DCs loaded with LAAs for post‐remission treatment of AML patients have been reported: vaccination with DCs electroporated with mRNA encoding *hTERT* resulted in antigen‐specific T‐cell responses in 11/19 patients; RFS after a median observation time of 52 months was 58%.^9^ Within a phase II trial, an anti‐leukaemic response was detected in 13/30 patients vaccinated with DCs loaded with *wilms tumor 1* (*WT1*) mRNA. A molecular remission defined by WT1 qPCR in the peripheral blood was achieved in 9/30 patients, and RFS and OS at 5 years were 30.8% and 50.0%, respectively.^10^ In both publications, DC maturation was achieved by a combination of pro‐inflammatory cytokines and prostaglandins.^11^ While this protocol was designed to promote migratory and immunostimulatory properties of DCs, no IL‐12p70 production was induced. However, IL‐12 is a crucial cytokine for both Th1 polarisation and NK cell activation. In preclinical work comparing DCs generated from peripheral blood mononuclear cells (PBMCs) of healthy controls using different maturation cocktails, we could show that the addition of a toll‐like receptor (TLR) 7/8 ligand to the DC maturation cocktail results in enhanced T‐cell stimulation. In direct comparison to DCs matured without a TLR agonist, the resulting DCs are characterised by a higher expression of the costimulatory molecules CD80 and CD86 and very high production of bioactive IL‐12p70. Both *in vitro* and *in vivo*, we could show that these DCs stimulate strong immune responses including polarisation of CD4^+^ T cells to Th1, induction of antigen‐specific CD8^+^ T cells and activation of NK cells.^12^, ^13^ This approach can be translated to monocytes derived from AML patients in CR, also resulting in IL12p70‐producing DCs with very similar functional characteristics.^14^

Hence, we have developed a good manufacturing practice (GMP)‐compliant protocol for the generation of next‐generation DCs, combining a short, only 3‐day differentiation period with a novel maturation cocktail that includes the TLR 7/8 agonist R848.^15^ As accounted for in detail previously^16^ mRNAs encoding the LAAs WT1 and preferentially expressed antigen in melanoma (PRAME) as well as the viral control antigen cytomegalovirus (CMV)pp65 were chosen for antigen loading of three separate batches of DCs by electroporation. Here, we describe the results of a phase I first‐in‐human proof‐of‐concept trial using next‐generation DCs for post‐remission therapy of 10 AML patients in first CR with a high risk of relapse (non‐favorable risk group or MRD positivity).

## RESULTS

### Patient characteristics

The characteristics of the 13 patients who were enrolled into the study are shown in Table 1. Twelve patients were positive for WT1 by qPCR at primary diagnosis, four were positive for PRAME by qPCR, and CMV serostatus was positive in four patients before vaccination. Eastern Cooperative Oncology Group (ECOG) performance status was 0 in two patients, 1 in 10 patients and 2 in one patient.

**Table 1:** Patient characteristics

	Gender	Age (years)	FAB	Cytogenetics	Molecular genetics	ELN risk group	Status of disease at SV1	WT1 expr prim dx	PRAME expr prim dx	CMV serostatus study start	ECOG	Leukocytes at dx (G L^−1^)	Tx prior DC vx
#1	m	72	M1	Complex karyotype	NPM1 wt CEBPA wt MLL neg	Adverse	CR	pos	neg	pos	1	2.6	s‐HAM, TAD‐9
#2	m	54	s‐AML (MDS)	del(12)(p13p13)(ETV6‐)	NPM1 wt FLT3‐ITD neg CEBPA wt MLL neg	Intermediate II	CRi	pos	pos	neg	1	3.7	s‐HAM, TAD‐9
#3	m	62	M4	Normal karyotype	NPM1 mut FLT3‐ITD+, FLT3‐TKD+ MLL neg	Intermediate I	beginning relapse	pos	pos	neg	1	93.9	s‐HAM, TAD‐9, AD, Vidaza
#4	f	48	M0	Normal karyotype	NPM1 wt CEBPA wt MLL neg, FLT3‐TKD‐, FLT3‐ITD‐, CEBPA wt	Intermediate I	beginning relapse	pos	neg	neg	1	0.9	s‐HAM, TAD‐9, AD
#5	f	44	M1	Normal karyotype	NPM1 wt CEBPA wt MLL neg	Intermediate I	beginning relapse	pos	neg	pos	1	1.6	s‐HAM, TAD‐9
#6	m	65	M2	Normal karyotype	NPM1 wt MLL‐PTD+, FLT3‐ITD, CEBPA wt	Intermediate II	CR	pos	neg	pos	1	13.9	7 + 3, HAM, 2 × HD‐Ara‐C
#7	f	74	M1	del(7q)	NPM1 wt FLT3 neg	Intermediate II	CR	neg	neg	neg	1	1.2	s‐HAM, TAD‐9
#8	f	79	s‐AML (MDS)	Normal karyotype	n.a.	Intermediate I	CR	pos	neg	neg	2	n.a.	Vidaza
#9	m	64	s‐AML (MDS)	Normal karyotype	NPM1 wt MLL‐PTD neg, FLT3 neg, CEBPA wt	Intermediate I	CR	pos	neg	neg	1	n.a.	s‐HAM, TAD‐9
#10	m	50	M1	Complex karyotype with inv(16)	NPM1wt, MLL‐PTD neg, inv16, FLT3‐ITD+, FLT3‐TKD+, CBFß‐MYH11 fusion transcript	Favorable	CR, MRD+	pos	pos	neg	1	75.1	AraC, sHAM, TAD‐9
#11	m	69	M1	inv(16)	NPM1 wt, FLT3‐ITD neg, FLT3‐TKD neg, MLL neg, CBFß‐MYH11 fusion transcript, inv16	Favorable	CRi, MRD+	pos	pos	neg	1	3.7	s‐HAM, TAD‐9
#12	m	55	M2	Normal karyotype	NPM1 wt, FLT3‐ITD neg, FLT3‐TKD neg, MLL‐PTD neg, CEBPA + mt	Intermediate I	CR	pos	neg	pos	0	2.8	s‐HAM, TAD‐9, AD, AC
#13	m	47	M0	Normal karyotype	NPM1 wt, FLT3‐ITD neg, FLT3‐TKD neg, MLL‐PTD neg, CEBPA wt	Intermediate I	CR	pos	neg	neg	0	1.6	s‐HAM, 3 days Fludarabin

AC, cytotoxic regimen consisting of cytarabine and cyclophosphamide; AD, cytotoxic regimen consisting of cytarabine and daunorubicin; CEBPA, CCAAT/enhancer‐binding protein alpha; CR, complete response; CRi, complete response with incomplete haematologic recovery; ECOG, Eastern Cooperative Oncology Group; ELN, European Leukemia Net; FAB, French–American–British classification; FLT3, fms‐like tyrosine kinase 3; ITD, internal tandem duplication; MLL, mixed‐lineage leukaemia; MRD, minimal residual disease; NPM1, nucleophosmin; s‐HAM, double induction regimen consisting of sequential high‐dose cytarabine and mitoxantrone; SV1, Screening Visit 1; TAD‐9, cytotoxic regimen consisting of thioguanine, cytarabine and daunorubicin; TKD, tyrosine kinase domain.

### Feasibility of vaccine generation and administration

Twelve patients underwent leukapheresis for production of the DC vaccine; patient #5 developed a leukaemia relapse in the short time span between screening and planned leukapheresis and was excluded from the study before leukapheresis. Key figures of the leukapheresis product are presented in Supplementary table 1. A median of 1.25 × 10^10^ (range 0.6–2.8 × 10^10^) viable white blood cells was collected per patient. Median monocyte yield was 3.6 × 10^9^ (range 1.0–7.5 × 10^9^). Median DC yield after electroporation was 3.65 × 10^8^ (range 1.27–5.68 × 10^8^). After quality control and removal of retain samples, sufficient DCs for the full schedule of 10 vaccinations (1.5 × 10^8^ DCs) were produced for 11 of 12 patients. For patient #2, only six vaccinations were available as the monocyte yield was low because of an unexpected decrease in leucocyte count between screening and leukapheresis (from 5.9 to 3.0 G L^−1^), and as DC recovery after electroporation was suboptimal. Two patients completed leukapheresis but were not vaccinated because of early relapse during vaccine production (#3) and because of characteristics of the vaccine (#8, see below). Of the 10 patients who actually initiated vaccination, seven underwent the complete regular schedule of 10 vaccinations. Patient #2 received all six vaccinations that were available, which was the minimum required by the study protocol; patient #4 developed a relapse after seven vaccinations and received two further vaccinations in combination with one cycle of 5‐azacytidine; and patient #7 also developed a relapse after seven vaccinations and received three further vaccinations in combination with two cycles of 5‐azacytidine. Two patients received further DC vaccinations after the end of the study in combination with 5‐azacytidine in view of an impending or established relapse: eight vaccinations with five cycles of 5‐azacytidine in patient #1 and two vaccinations with one cycle of 5‐azacytidine in patient #11. Median time from CR/CR_i_ to first vaccination was 110 days (range 34–205 days), mainly because of further cycles of consolidation therapy; median time from leukapheresis to first vaccination was 25 days (range 18–38 days).

### Vaccine characterisation

**Figure 1:**
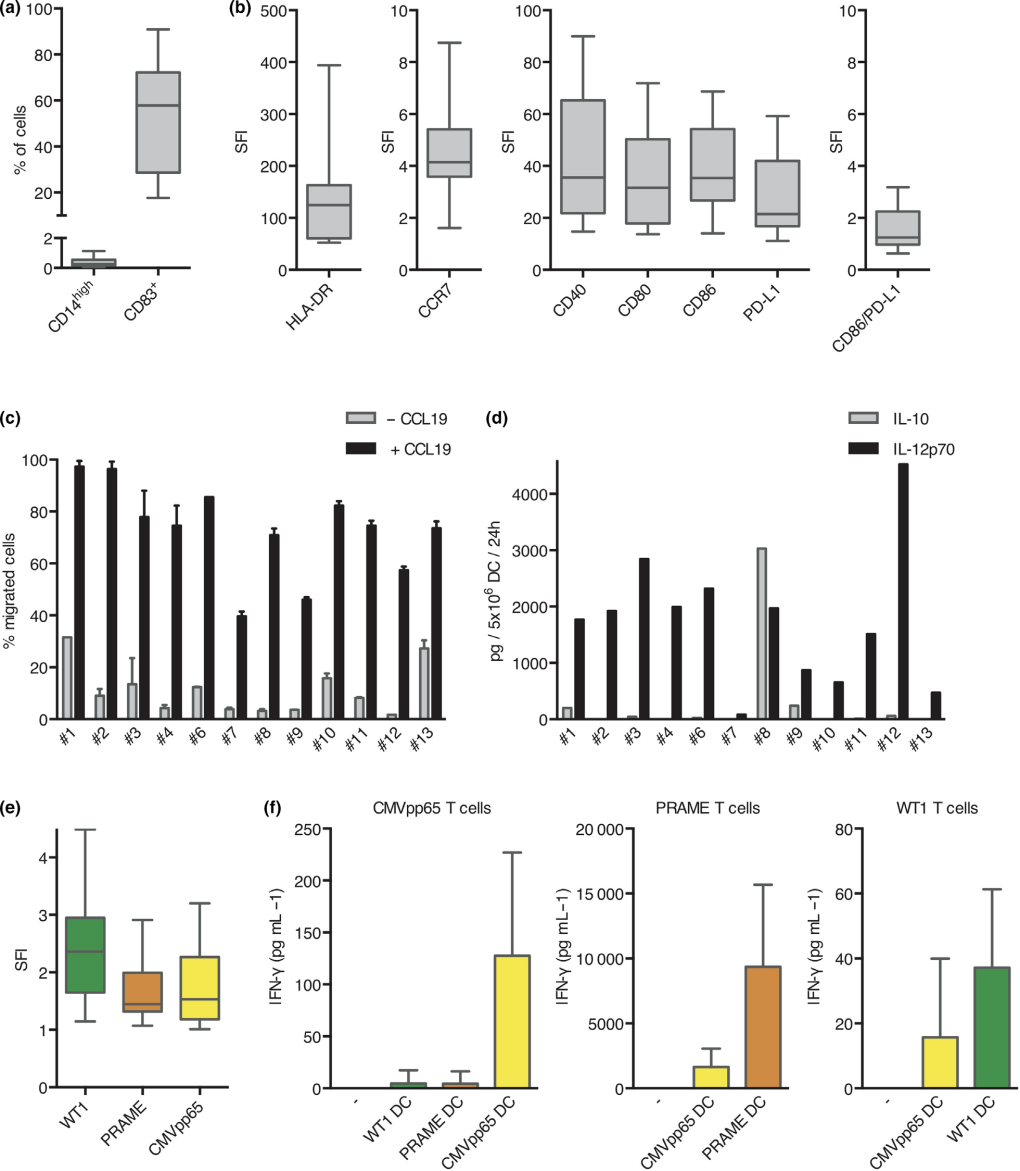
Characterisation of DC phenotype, migration capacity, cytokine secretion and antigen processing and presentation. For all 12 generated DC preparations, surface expression of **(a)** the DC markers CD14 and CD83 and **(b)** various costimulatory or chemokine receptor molecules was determined by flow cytometry. **(c)** Migration towards a CCL19 gradient was measured in a trans‐well assay (2 technical replicates per sample). **(d)** Secretion of IL‐10 and IL‐12p70 after CD40 ligation was analysed. To prove successful antigen translation and presentation after RNA electroporation, DCs were **(e)** intracellularly stained for the resulting proteins and **(f)** used for stimulation of specific T‐cell clones as measured by IFN‐γ secretion (*n* = 3–7). For **a**, **b** and **e**, results are presented in box‐and‐whisker plots, with boxes representing the lower quartile, the median and the upper quartile, while the whiskers show the minimal and the maximal values. For all other graphs, data shown reflect mean and standard deviation.

All 12 generated DC preparations were tested for their phenotype, migration capacity, cytokine secretion, and processing and presentation of the three selected antigens after RNA electroporation (Figure 1 and Supplementary figure 1). For all patients, the cells showed a typical DC phenotype (CD14^low^ and CD83^+^; Figure 1a). Expression of various costimulatory or chemokine receptor molecules was measured, and the specific fluorescence intensity (SFI) was calculated (Figure 1b). Median SFI was 124.6 for HLA‐DR, 4.1 for CCR7, 35.6 for CD40, 31.6 for CD80, 35.4 for CD86, and 21.5 for PD‐L1. The ratio of CD86 to PD‐L1 expression as a potential measure of positive costimulation was 1.25 in median. A median of 74.5% (range 38.3–98.4%) of DCs showed migration towards a CCL19 gradient (Figure 1c). Ten of 12 DC preparations secreted relatively high amounts of IL‐12p70 (median of 1845 pg/5 × 10^6^ DC/24 h; range 470–4525 pg/5 × 10^6^ DC/24 h) and low amounts of IL‐10 (median of 17.3 pg/5 × 10^6^ DC/24 h; range 0–241 pg/5 × 10^6^ DC/24 h), as expected from our previous experiments.^12^ DCs of patient #7 showed very low IL‐12p70 production (81.5 pg/5 × 10^6^ DC/24 h) and no IL‐10 production. DCs of patient #8 showed high IL‐12p70 production (1969 pg/5 × 10^6^ DC/24 h), but even higher IL‐10 production (3031 pg/5 × 10^6^ DC/24 h; Figure 1d). Because of the unknown effects of vaccinations with IL‐10‐producing DCs in the AML setting, this patient was excluded from the study and not vaccinated, although all release criteria for the vaccine were fulfilled. Successful translation of the electroporated RNA was proven by intracellular staining of the DCs for the resulting proteins (median SFI 2.36 for WT1, 1.44 for PRAME, 1.53 for CMVpp65); DCs electroporated with one of the other two RNA molecules served as control (Figure 1e and Supplementary figure 2). Presentation of the antigens in the context of HLA molecules was functionally proven by IFN‐γ secretion of specific T‐cell clones after coculture with the different DC batches. Each T‐cell clone was preferentially stimulated by the respective DC batch (Figure 1f).

### Vaccine‐induced immune responses

**Figure 2:**
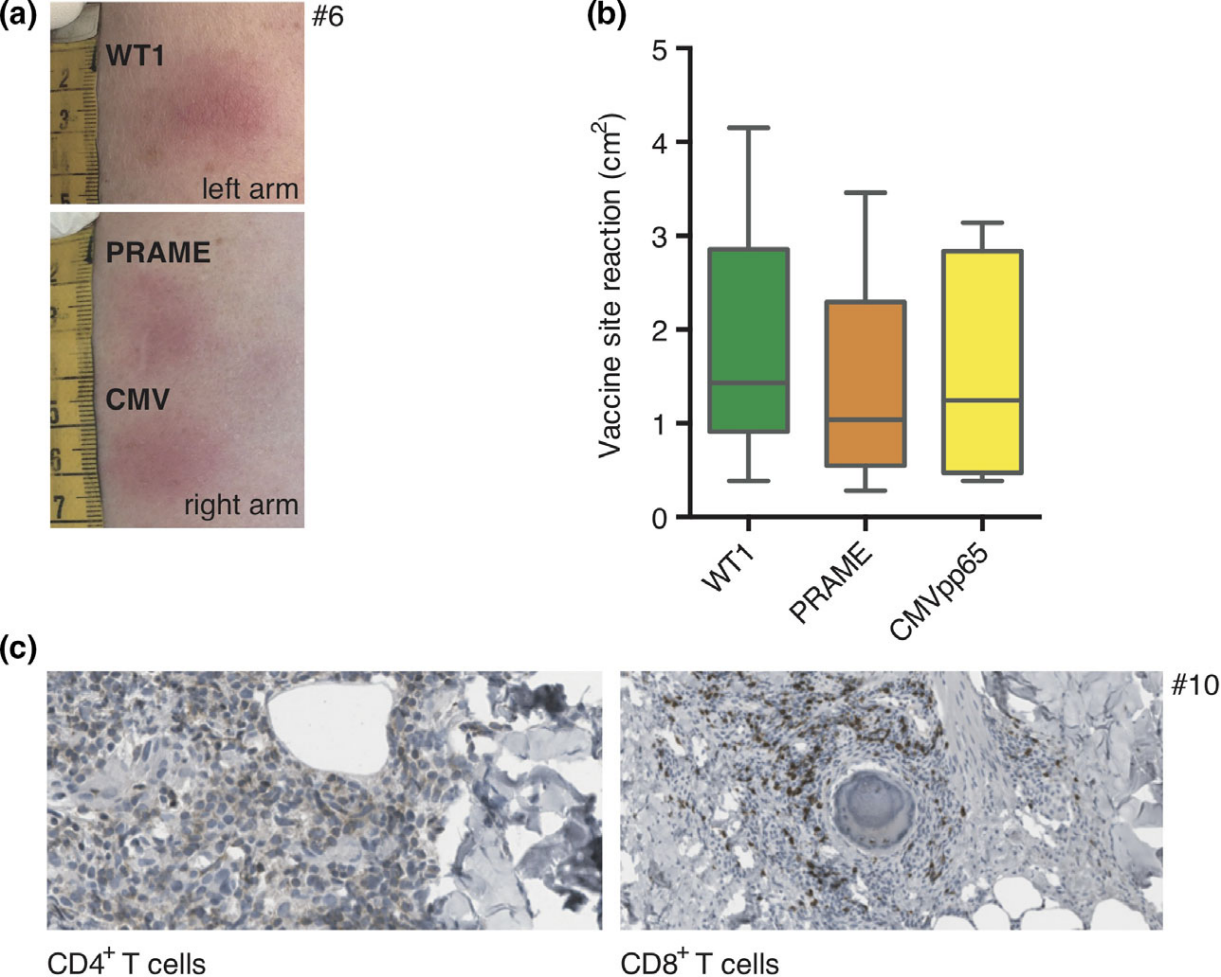
Vaccine site reaction. **(a)** For all 10 vaccinated patients and all antigens, erythema and induration of the vaccine sites were observed. **(b)** There was high variability between patients, but no significant difference between the three antigens in size of local reaction. **(c)** Immunohistochemical analysis of skin biopsies at the vaccine sites revealed dense CD4^+^ and CD8^+^ T‐cell infiltration (one representative example shown).

For all 10 vaccinated patients, local immune response was measured 48 h after the fifth vaccination by size of local erythema and induration (Figure 2a). Vaccine site reaction was detectable for all patients and all antigens. Variability between patients was high, but no significant differences were found between the three antigens (WT1: median of 1.43 cm^2^, range 0.38–4.15 cm^2^; PRAME: median of 1.04 cm^2^, range 0.28–3.46 cm^2^; CMV: median of 1.24 cm^2^, range 0.38–3.14 cm^2^; Figure 2b). Skin biopsies were taken from nine patients. Dense CD4^+^ and CD8^+^ T‐cell infiltration was seen by immunohistochemistry (Figure 2c).

**Figure 3:**
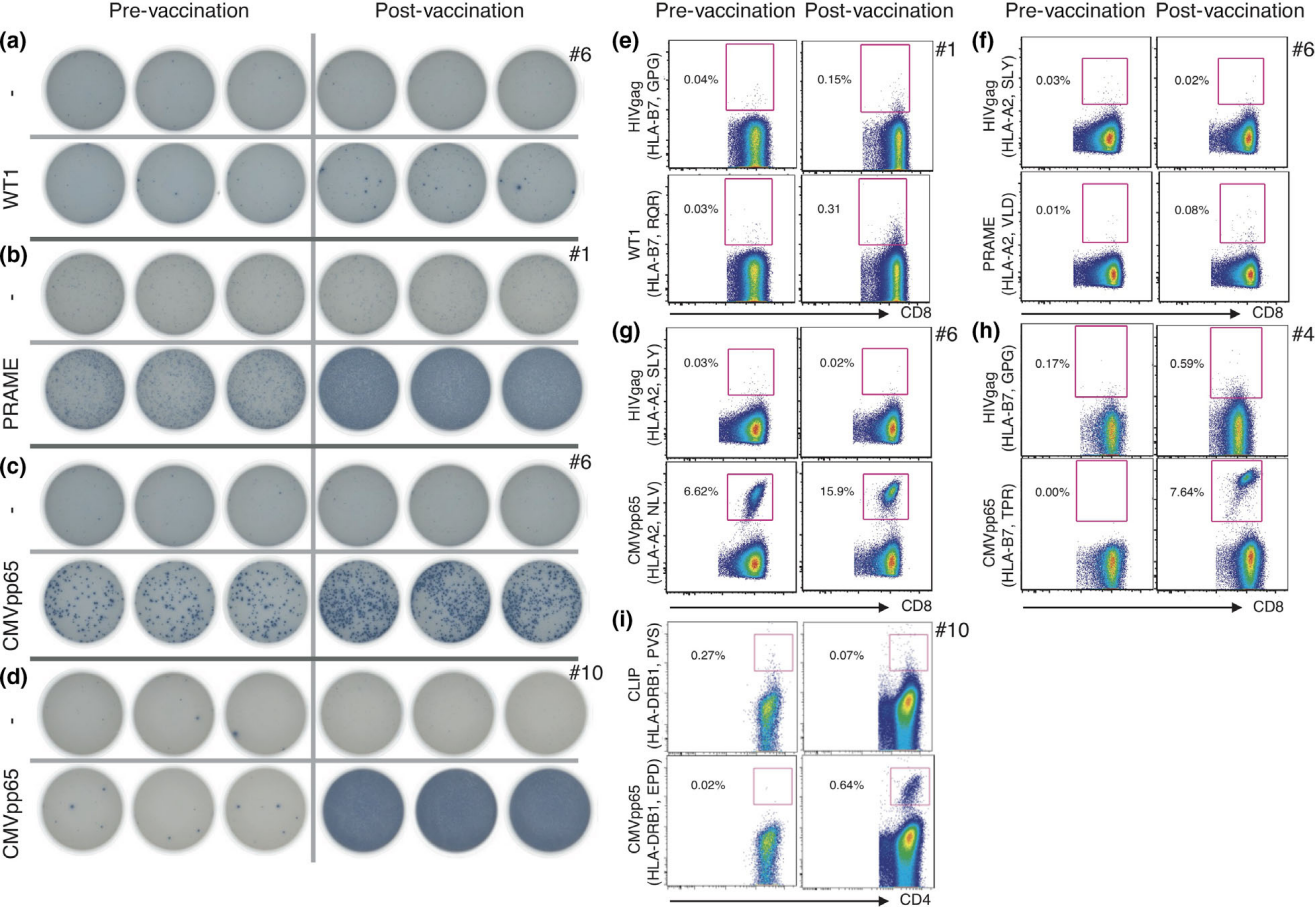
Representative examples of vaccine‐induced immune responses. **(a–d)** PBMCs isolated before and after vaccination were tested for antigen‐specific T cells by ELISpot. Increased immune responses were detected for the LAAs WT1 **(a)** and PRAME **(b)** as well as for CMVpp65 **(c, d)**. Both expansion of pre‐existing immune responses **(c)** and induction of novel immune responses **(d)** were observed. **(e–h)** PBMCs isolated before and after vaccination were tested for antigen‐specific CD8^+^ T cells by multimer staining. Increased immune responses were detected for the LAAs WT1 **(e)** and PRAME **(f)** as well as for CMVpp65 **(g, h)**. Both expansion of pre‐existing immune responses **(g)** and induction of novel immune responses **(h)** were observed. **(i)** For CMVpp65, induction of antigen‐specific CD4^+^ cells was also detected.

Immunomonitoring was performed on PBMCs and plasma samples obtained before vaccination, after five vaccinations and at the end of the study. We found no major changes in the course of the therapy with respect to absolute and relative numbers of leucocytes, granulocytes, monocytes, lymphocytes, CD3^+^ T cells, CD4^+^ T cells, CD8^+^ T cells, CD19^+^ B cells or CD3^‐^/CD16_56^+^ NK cells (data not shown). Antigen‐specific T‐cell responses were measured by ELISpot and by multimer staining, as shown for representative patients in Figure 3 (complete immunomonitoring data of these patients is presented in Supplementary figure 3). An increased ELISpot response after vaccination as defined by a ≥ 1.5‐fold increase of antigen‐specific spot count was detected in 2/10 patients for WT1 (Figure 3a), in 4/10 patients for PRAME (Figure 3b), and in 9/10 patients for CMV (Figure 3c and d; Table 2). These results were largely reflected by multimer staining: an increased response as defined by a ≥ 2‐fold increase of multimer‐positive CD8^+^ T cells was detected in 1/6 patients for WT1, in 0/3 patients for PRAME, and in 6/8 patients for CMV, with limitations because of the availability of multimers for the various HLA types (Table 2 and Supplementary figure 4). CMV responses were generally very high, with up to 15.9% of all CD8^+^ T cells stained with a single CMV multimer after vaccination in a primarily seropositive patient (#6; Figure 3g), and up to 9.6% of all CD8^+^ T cells stained with a single CMV multimer after vaccination in a primarily seronegative patient (#10). Of note, also decreased frequencies after vaccination were observed (Supplementary figure 3). Post‐vaccination LAA‐specific T‐cell responses were significantly lower, but still clearly detectable in some patients (Figure 3e and f). In 4/7 patients where a CMV‐specific multimer for HLA type II was available, an increase in antigen‐specific CD4^+^ T cells could be detected as well (Figure 3i; Table 2).

**Table 2:** Immune responses to the study antigens

Pt	Immune responses	ELISpot						Multimer						
		WT1		PRAME		CMVpp65		MHC	WT1		PRAME		CMVpp65	
		Prior vx	Post vx	Prior vx	Post vx	Prior vx	Post vx		Prior vx	Post vx	Prior vx	Post vx	Prior vx	Post vx
#1	WT1/PRAME/CMV	−	↑	++	↑	++	↑	I	−	↑			++	↓
								II					++	↓
#2	CMV	−	=	−	=	−	↑	I	−	=	−	=	−	↑
								II					−	=
#4	CMV	−	=	−	=	−	↑	I	−	=			−	↑
								II					−	↑
#6	WT1/PRAME/CMV	−	↑	+	↑	++	↑	I	+	=	−	↑	++	↑
								II						
#7	CMV	−	=	−	=	−	↑	I						
								II					−	↑
#9	PRAME/CMV	−	=	+	↑	−	↑	I						
								II						
#10	PRAME/CMV	−	=	−	↑	−	↑	I					−	↑
								II					−	↑
#11	–	−	=	−	=	−	=	I	−	=	+	↓	−	=
								II					−	=
#12	CMV	−	=	−	=	++	↑	I					++	↑
								II					−	↑
#13	CMV	−	=	−	=	−	↑	I	+	=			−	↑
								II						

Prior vaccination (vx): −, no immune response; +, pre‐existing immune response; ++, strong pre‐existing immune response. Post vx: ↑, increase in multimer‐positive T cells or ELISpot response; =, no increase or decrease in multimer‐positive T cells or ELISpot response; ↓, decrease in multimer‐positive T cells or ELISpot response. For definitions, see Methods.

Vaccine‐induced B‐cell responses were measured by detection of CMV antibodies. Of seven patients who were CMV seronegative before vaccination, antibodies against CMV were detected in three patients after vaccination (#7, #10, #13), and one patient had a borderline reaction after vaccination (#2), while no antibodies against CMV were detectable in three patients (#4, #9, #11). Seroconversion as a result of primary CMV infection was excluded by the methodology.

### Clinical responses to vaccination

The vaccination protocol was generally very well tolerated. All patients observed transient vaccine site reactions (erythema, induration, pruritus) of grade 1 intensity. Other frequent adverse events were musculoskeletal pain (6/10), skin reactions outside of vaccine sites (5/10), diarrhoea (4/10) and fatigue (4/10). All potentially treatment‐related adverse events reported by ≥ 2/10 patients are listed in Supplementary table 2. All adverse events were transient, and except for one grade 3 pyrexia, all adverse events were graded 1–2.

**Figure 4:**
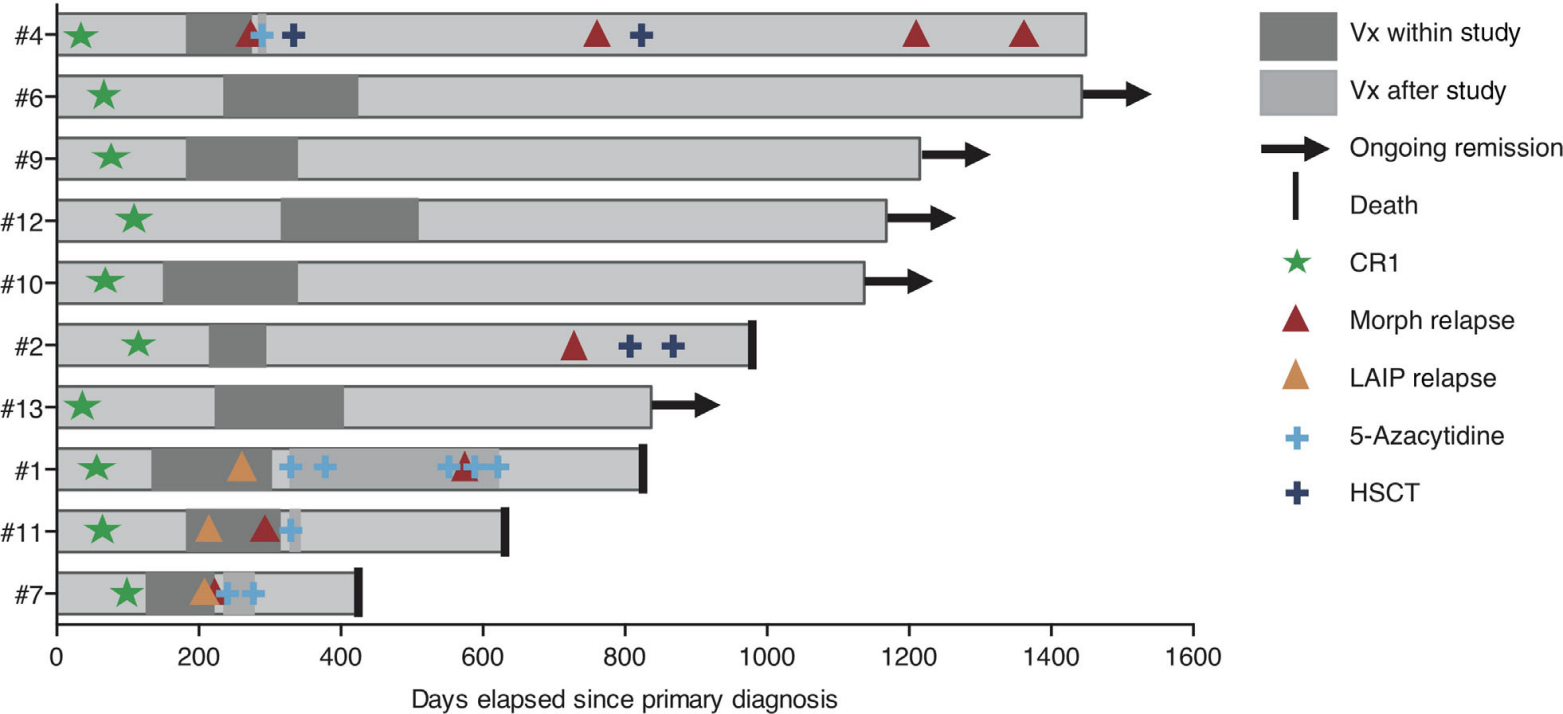
Swimmer plot. Time point of first CR, vaccinations, potential other treatment modalities, and relapses, death or ongoing remission are depicted for all patients treated within the trial.

**Figure 5:**
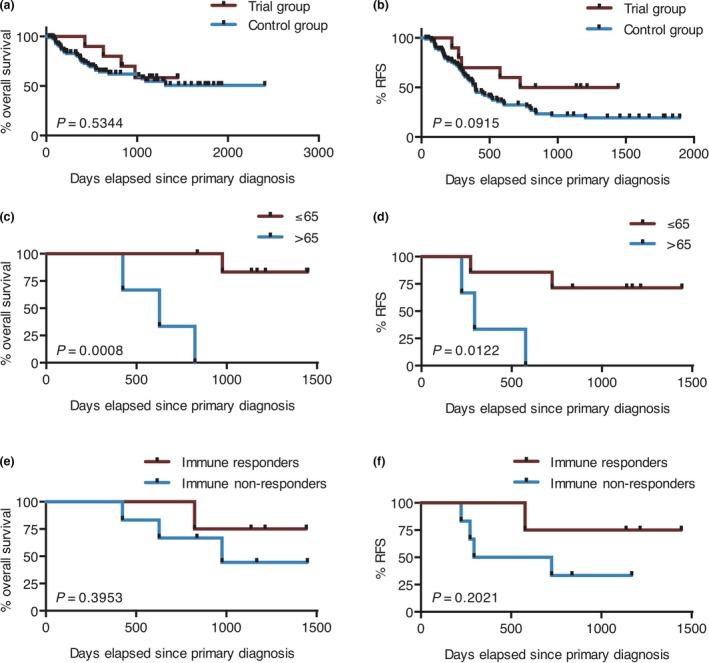
Survival analysis. OS **(a, c, e)** and RFS **(b, d, f)** of the vaccinated patients were depicted by Kaplan–Meier plots and compared by the log‐rank test. **(a, b)** Patients treated within the trial were compared to a closely matched cohort of 88 patients from the AML‐CG registry. **(c, d)** Within the study cohort, patients ≤ 65 years and > 65 years at time of diagnosis were compared. **(e, f)** Immune responders as defined by an increase in LAA‐specific T cells after vaccination were compared to immune non‐responders.

Because of limited patient numbers in the phase I setting, clinical efficacy analysis was purely exploratory. Vaccinated patients have been observed for a median of 1057 (range 424–1449) days since primary diagnosis and a median of 811.5 (range 293–1267) days since first vaccination, with the cut‐off on 31 March 2018. A swimmer plot of all 10 vaccinated patients is depicted in Figure 4. Three patients (#4, #7 and #11) relapsed already in the course of the scheduled vaccinations, and two patients (#1 and #2) relapsed after the end of the trial. Of these five patients, only one (#4) is still alive after several salvage therapies. The other five vaccinated patients are still alive and in ongoing CR. Aggregated survival data are shown in Figure 5. Median OS has not yet been reached (Figure 5a), and median RFS was 1084 days (Figure 5b), with 50% of patients still relapse‐free at the end of observation. In a hypothesis‐generating analysis, these survival data compare favorably to a closely matched patient cohort from the AML‐Cooperative Group (AML‐CG) registry (see Supplementary table 3 for patient characteristics), where median OS was also not yet reached at the end of observation (*P*‐value = 0.53; Figure 5a) and median RFS was only 396 days, closely missing out on statistical significance in spite of the small trial group (*P*‐value = 0.09; Figure 5b). Exploratory subgroup analysis within the study cohort showed that patients ≤ 65 years had significantly better OS (median not yet reached vs. 628 days; *P*‐value = 0.0008; Figure 5c) and RFS (median not yet reached vs. 294 days; *P*‐value = 0.0122; Figure 5d) than patients > 65 years. Immune responders as defined by expansion of antigen‐specific T cells against WT1 or PRAME showed a trend towards better OS (median not yet reached vs. 976 days; Figure 5e) and RFS (median not yet reached vs. 509 days; Figure 5f) than immune non‐responders, but statistical significance was not reached because of the low patient number. Specifically, the three patients ≤ 65 years who showed an LAA‐specific immune response (#6, #9 and #10) are all in ongoing CR.

### Combination of 5‐azacytidine with DC vaccination as individual treatment attempt

Towards the end of the study treatment, patient #1 developed an increase in MRD load, for both WT1 copy number and frequency of leukaemia‐associated immunophenotype (LAIP), predicting an impending relapse (Supplementary figure 4a). After positive discussion with the ethics committee of the LMU Munich and written informed consent by the patient, we started an individual treatment attempt combining 5‐azacytidine in the approved dose and schedule (75 mg m^−2^ s.c. on days 1–7 of a 28‐day cycle) with next‐generation DC vaccination on day 8 and day 15 (Supplementary figure 4a). Vaccine site reactions were found to be considerably enhanced (Supplementary figure 4b), and the frequency of LAA‐specific T cells was increased (Supplementary figure 4c). Two cycles of this combination therapy lead to MRD conversion (Supplementary figure 4a), which lasted for some time before the patient relapsed almost a year later. Similar treatment attempts were later repeated for patients #4, #7 and #11, however not in MRD situation, but in overt relapse. Similar results in terms of local reaction and reduction of disease burden were not observed in these cases.

## Discussion

As detailed above, two clinical trials using monocyte‐derived DCs loaded with LAA‐specific mRNA have already been published.^9^, ^10^ In both studies, DCs were activated by the classical combination of pro‐inflammatory cytokines and prostaglandins,^11^ and mRNA encoding a single LAA (*hTERT* and *WT1*, respectively) was used for electroporation. Our trial decisively differed in two important respects. First, the TLR7/8 ligand R848 was included into the maturation protocol, resulting in DCs with improved immunostimulatory properties including secretion of IL‐12p70, as demonstrated in detail previously.^12^ This study represents the first‐in‐human trial applying these next‐generation DCs to patients. Second, three antigens were chosen for loading of separate DC batches.^16^ Next to WT1, which is very frequently overexpressed in AML and the most prominent antigen in vaccination trials for AML, both for DC vaccination^10^, ^17^, ^18^ and for peptide vaccination,^5^ we decided to add a second LAA in order to broaden anti‐leukaemic responses and to decrease the possibility of immune escape. We chose PRAME as the most prominent cancer–testis antigen in AML.^19^, ^20^ CMVpp65 as a very abundant and immunogenic viral antigen was added for loading of a third batch of DCs, allowing us to differentiate between the induction of primary and secondary immune responses by comparison of CMV‐seronegative and CMV‐seropositive patients.

The primary objective of this trial using next‐generation DCs for post‐remission therapy of AML patients was to explore the feasibility of DC generation as well as the safety of the vaccinations. Patients in first CR after intensive chemotherapy, but with a high risk of relapse, could be included. Three of the 13 patients who were enrolled did not proceed to vaccination because of disease‐related factors (very early relapse prior to the first vaccination; *n* = 2) or because of factors related to vaccine production (high IL‐10 secretion by DCs; *n* = 1). The high production of IL‐10 by the DC vaccine produced for patient #8 was unique and had never been seen before in preclinical experiments. This accentuates both the very high relapse risk of the enrolled patients and the high success rate (> 90%) in production of DCs secreting high amounts of IL‐12p70 and low amounts of IL‐10. For the other 10 patients, the generated DCs sufficed for vaccination of all three antigens at the minimum of six specified time points. Median time between leukapheresis and start of the vaccination was 25 days (Supplementary table 1). Eight of these 10 patients completed the full study protocol, while two were taken off study because of early relapse, again highlighting the unfavorable prognosis of the included patients. We conclude that generation and administration of next‐generation DCs are feasible in AML patients after intensive chemotherapy, albeit early relapse can prevent successful administration in very high‐risk patients. Judging from the 105 vaccinations that were administered in total, tolerability of the protocol was excellent. Only transient adverse events were observed, and except for one grade 3 pyrexia, all adverse events were graded 1–2 (Supplementary table 2). Despite using DCs with stronger immunostimulatory capacity compared to prior vaccination studies, our data showed an excellent safety profile.

As a secondary objective of the trial, we studied immunological responses to the DC vaccinations. Antigen loading was done by electroporation of mRNA in order to allow for HLA‐independent, multiple‐epitope antigen presentation. T‐cell responses before and after the vaccinations were detected by multimer staining and by ELISpot. The analysis of CMVpp65‐specific T‐cell responses allowed us to distinguish between T‐ and B‐cell responses in latent CMV carriers in comparison with CMV negative patients. Within our cohort, 3/10 patients were seropositive for antibodies against human CMV. Before the vaccinations, we detected antigen‐specific T cells by tetramer and ELISpot in all three seropositive patients (Table 2). Interestingly, we observed an induction of a T‐cell response to CMVpp65 in all but one patient after vaccination, and an expansion of CMVpp65‐specific T cells in seropositive patients. For one patient (#1), we observed divergent results between ELISpot and multimer assays, with strong upregulation of the ELISpot response and downregulation of the multimer‐positive population. We hypothesise that this might be interpreted as a selective expansion of antigen‐specific T cells not detected by the available multimers or possible determinant spreading to T cells recognising an alternative epitope. Using major histocompatibility complex (MHC) class II multimers, we found an increase in antigen‐specific CD4^+^ T cells in four of seven applicable patients (Table 2). In two of these patients (#7, #10), this correlated with development of antibodies against CMVpp65. A physiological seroconversion as a result of primary CMV infection was ruled out by missing detection of the CMV‐associated protein p150. From the data on CMVpp65 immunomonitoring, we conclude that next‐generation DCs are capable of inducing primary and secondary immune responses. These are not restricted to CD8^+^ T‐cell responses, but also comprise CD4^+^ T‐cell and antibody responses.

Similarly, we were able to show the induction of LAA‐specific T‐cell responses. However, in contrast to the immune responses against CMVpp65, the responses directed against WT1 and PRAME were lower in frequency and not detected in all patients. This might partially be attributed to restricted availability of HLA‐specific multimers and a random mix of peptides with different lengths for the ELISpot assays. Therefore, it is likely that not all LAA‐specific T cells were detected in spite of the two complementary methods. However, differences between a viral antigen and autoantigens certainly play a role, with high‐affinity T cells against the latter being negatively selected in the thymus during T‐cell development. Our immunomonitoring data provide evidence that the immunostimulatory capacity of next‐generation TLR7/8‐matured DCs is very high. Further work is needed to identify the optimal setting for DC application, for example induction of neoantigen‐specific T cells or boosting of genetically engineered T cells for adoptive transfer.

In spite of the single‐arm phase I design and the limited patient number in this first‐in‐human trial, we believe it is highly relevant to report the safety and tolerability of a TLR7/8‐matured DC vaccine. The successful application of more than 100 vaccines demonstrates the suitability of the vaccine, which is also applicable in other cancer entities. Besides, we demonstrated the induction of immunological responses. Promising clinical outcome is suggested by the comparison to a closely matched patient cohort. A beneficial effect of vaccination was observed with respect to RFS with a median survival of 1084 compared to 396 days. This effect was more pronounced for patients of younger age and with vaccine‐induced immune responses. Patients ≤ 65 years showed significantly better OS and RFS than patients > 65 years. Two of the patients in the older cohort relapsed quickly without detection of a LAA‐specific immune response, and the third patient relapsed shortly after termination of the vaccination protocol. In the younger cohort, however, only two of seven patients relapsed, and six of seven were still alive at data cut‐off. This is in line with a recent publication, in which an overall survival benefit was dominantly observed in the patient cohort below 65 years of age.^10^ This might be related to the larger pool of naive T cells in younger AML patients, which are required for the induction of novel anti‐leukaemic immune responses.^21^ Moreover, immune responses against WT1 and PRAME correlated to prolonged OS and RFS (Figure 5e and f). Specifically, all three patients of the younger age group that showed a leukaemia‐specific immune response remained in ongoing CR until data cut‐off. Our data support the hypothesis that TLR7/8‐matured DCs induce protective LAA‐specific immune response in patients ≤ 65 years. However, frequency and strength of LAA‐specific immune responses need to be enhanced in order to improve clinical benefit.

Of note, because of the very small patient number, the comparison of survival data with the matched patient cohort is purely exploratory and hypothesis‐generating. There was therefore no formal statistical analysis plan for this comparison, and multiple testing was not compensated for.

Several factors might have contributed to the fact that the immunological and clinical effects in this study were lower than might have been expected. The use of autoantigens for vaccination has been discussed above. Second, a comparison of the DC characterisation within this trial with the results of our preclinical experiments^12^, ^13^, ^15^ showed considerably lower CD86/PD‐L1 ratio and IL‐12p70 secretion. This might be due to the upscaling of the DC generation process including elutriation of a leukapheresis product after overnight storage instead of plastic adherence of freshly isolated PBMCs.

However, we believe that combinatorial approaches are the most promising strategy to further enhance immune responses and hence clinical benefit. Epigenetic modifiers such as DNA methyltransferase inhibitors and histone deacetylase inhibitors are suitable combination partners because of an enhancement in antigen processing and presentation of malignant cells.^22^, ^23^, ^24^, ^25^ In the setting of myelodysplastic syndrome, the combination of vaccination against NY‐ESO‐1 and decitabine resulted in an increased antigen‐specific immune response.^26^ In our hands, the combination of next‐generation DC vaccination with 5‐azacytidine resulted in a striking increase in local and systemic immune responses. This translated into a temporary MRD conversion in a single patient. We suggest pursuing this approach in further clinical trials. Immune checkpoint blockade is another strategy for combinational approaches. Early clinical trials are already combining vaccines with programmed cell death protein 1 (PD‐1) blockers for treatment of various malignancies including AML.^27^ The combination of both epigenetic modification by azacytidine and PD‐1 blockade by nivolumab was recently shown to be a safe and effective therapy for relapsed AML.^28^ However, other checkpoint molecules might be even more relevant as suggested by our preclinical data showing that blockade of lymphocyte activation gene 3 (LAG‐3) strongly enhances DC‐induced immune responses against viral and leukaemia‐associated antigens.^29^

## Conclusions

Vaccination of high‐risk AML patients with TLR7/8‐matured RNA‐loaded DCs was feasible, safe and resulted in induction of leukaemia‐specific immune responses. Explorative comparison to a matched cohort suggests a benefit on the clinical outcome; positive effects of vaccination on survival were particularly seen for immune responders and patients ≤ 65 years. Perspectively, immune responses can be further augmented by combining TLR7/8‐matured DCs with immunomodulatory drugs like hypomethylating agents or checkpoint inhibitors.

## Methods

### Study design

We here report results of a phase I trial, with clinical efficacy analysis being purely explorative. AML (excluding acute promyelocytic leukaemia) patients at the age of 18–75 with a non‐favorable risk profile (intermediate I, intermediate II or adverse according to European LeukemiaNet (ELN) classification of 2010;^30^ or with a favorable risk according to ELN and MRD positivity) in CR/CR_i_ after at least one cycle of intensive induction therapy including an anthracycline and cytarabine were eligible for enrolment. Patients with prior allo‐HSCT, severe organ dysfunction or active clinically relevant autoimmune disease were excluded. None of the patients were eligible for an allo‐HSCT, either because of comorbidities, lack of donor or missing consent. The primary objective of the study was to determine safety and feasibility of immunotherapy with autologous DCs, resulting in the endpoints of frequency of adverse events and percentage of patients in whom treatment with the scheduled number of immunotherapies (10 DC vaccinations) was feasible. As a secondary objective, we explored the induction of immunological responses to the DC vaccination. Clinical responses were estimated by comparing RFS and OS between immune responders and non‐responders as well as between all vaccinated study patients and matched control patients of the AML‐CG registry. The vaccine was administered intradermally up to 10 times within 26 weeks at 5 × 10^6^ DCs for each antigen (three batches at three separate sites) and time point, starting at weekly intervals and continuing at four‐week intervals (see Supplementary figure 5). No other anti‐leukaemia therapy was permitted in parallel as long as the patient was in remission, but 5‐azacytidine was added to the ongoing vaccination strategy in some patients when the criteria for a leukaemia relapse were met. The study was mono‐centric, open‐label, prospective and non‐randomised. All patients with successful vaccine generation who still met the eligibility criteria after this process were vaccinated at the Department of Medicine III, University Hospital, LMU Munich.

### Vaccine generation

Peripheral blood mononuclear cells were collected by leukapheresis and transported to the GMP facility of the Department of Cellular Therapy at The Norwegian Radium Hospital in Oslo. Monocytes were enriched from leukapheresis using elutriation (ELUTRA, Caridian) and cultured in RPMI 1640 medium with very low endotoxin (Biochrom, Berlin, Germany) plus 1.5% human AB serum (Institute of Transfusion Medicine, Suhl, Germany), supplemented with 560 IU mL^−1^ GM‐CSF (Leukine^®^, Bayer, Leverkusen, Germany) and 20 ng mL^−1^ interleukin‐4 (R&D Systems, Wiesbaden, Germany) for 40–72 h. Thereafter, 10 ng mL^−1^ TNF‐α, 10 ng mL^−1^ IL1‐β (both R&D Systems, Wiesbaden, Germany), 5000 IU mL^−1^ interferon‐γ (Imukin^®^, Boehringer Ingelheim, Ingelheim, Germany), 250 ng mL^−1^ PGE2 (Prostine^®^ E2; Pfizer, Kent, UK) and 1 μg mL^−1^ R848 (3M Pharmaceuticals, St. Paul, MN, USA) were added to the culture medium for another 20–26 h.^16^ Mature DCs were thoroughly washed and electroporated in three different batches, each transduced with *in vitro* transcribed (ivt) codon‐optimised RNA (produced at Oslo University Hospital in clinical grade) encoding for either human *WT1* (isoform A, NP_000369.3), *PRAME* (NP_006106.1) or *CMVpp65* (P06725.2). After 2–6 h, DCs were harvested and cryopreserved. Before the first batch of DCs was administered to the individual patient, release criteria including total cell number, viability, and CD80 positivity, as well as lack of excessive contaminating cells, microbiological contamination and mycoplasma, were controlled (see Supplementary table 5 for details). Before administration, cells were resuspended with 200 µL DPBS each.

### Vaccine characterisation

Expression of DC surface antigens was measured by flow cytometry using a panel of fluorescence‐conjugated monoclonal antibodies (Supplementary table 6). Dead cells were excluded by Live/Dead Aqua (Invitrogen, Carlsbad, CA, USA) staining and only singlets gated. Corresponding mouse IgG isotype controls were used. After washing, cells were analysed using a FACS LSR II (BD Biosciences). Post‐acquisition analysis was performed using FlowJo software (version 9.7.6; Tree Star, Ashland, OR, USA). The percentage of positive cells was determined by setting the gate at or below 1% in the respective isotype control. SFI was calculated as the ratio of the median fluorescence intensity of the test sample to its corresponding isotype control. Migration and cytokine secretion capacity of DCs were analysed as described previously.^14^ To assess protein expression of transfected RNA in DCs, the freshly thawed cells were fixed using Foxp3 Staining Buffer Set (eBioscience). After FcR blocking, intracellular antigen staining was performed with anti‐HCMV, anti‐WT1 or anti‐PRAME, and AF647‐conjugated anti‐mouse F(ab)2 as secondary antibody (Supplementary table 6). DC antigen presentation capacity was tested in an human leucocyte antigen (HLA)‐matched 24h coculture of *CMVpp65*, *WT1* or *PRAME* RNA‐transfected DCs with CMV‐specific T cells (kindly provided by A. Moosmann), WT1‐specific T cells (generated in our laboratory as previously described^31^) or PRAME‐specific T cells (generated as previously described^32^), respectively, at a 1:10 ratio. IFN‐γ secretion into the supernatant was analysed by cytometric bead array (CBA) Human IFN‐γ Flex Set (BD Biosciences).

### Measurement of immune responses

Local reactions at the vaccine sites were assessed by measuring the diameter of the erythema 48h after the fifth vaccination. Skin biopsies were taken and analysed by immunohistochemistry for CD4^+^ and CD8^+^ T‐cell infiltration. Patients' lymphocyte subpopulations in peripheral blood were analysed according to standard procedures. Human IFN‐γ single‐colour ELISpot assays (CTL, Bonn, Germany) were performed following the manufacturer's recommendations with 2µg mL^−1^ CMVpp65, WT1 or PRAME peptide pools (JPT, Berlin, Germany) in triplicates. Resulting spots were counted using the ImmunoSpot S6 Analyzer's (CTL) Smart Count Mode. Multimer staining was performed depending on the patient's HLA (Supplementary table 4) and availability of corresponding multimers. PE‐labelled multimers (Supplementary table 6) were used for identification of vaccine‐induced CD4^+^ and CD8^+^ T cells specific for CMVpp65, WT1 and PRAME. Multimers for HIV‐Gag and CLIP were used as controls. For detection of CMV‐specific CD4^+^ T cells by MHC class II multimers, PBMCs were expanded for 7 days in the presence of 2.5 µm CMVpp65 peptide EPDVYYTSAFVFPTK (JPT) with 5 ng mL^−1^ IL‐7 und IL‐15 (PeproTech) added during the last three days. T‐cell surfaces were additionally stained for CD3, CD4 and CD8. Patient sera were analysed for antibodies against the single antigens of human CMV before and after vaccination using the recomLine CMV IgG, IgM Immunoassay (MIKROGEN, Neuried, Germany) and the Enzygnost^®^ (Siemens Healthcare GmbH, Erlangen, Germany). Primary CMV infection during the trial was excluded by assessment of the study‐specific p65 protein without concomitant detection of the p150 protein.

### Clinical assessments

Patients were monitored for adverse events starting from the first screening visit until 4 weeks after the last vaccination. All toxicities were graded according to the National Cancer Institute Common Toxicity Criteria version 5.0. Leukaemia was assessed by routine bone marrow diagnostics including determination of MRD by available molecular markers and by LAIP. RFS and OS were followed until the cut‐off date of 31 March 2018 and depicted by swimmer plot for individual patients and by Kaplan–Meier plots.

### Statistical analysis

For the analysis of ELISpot responses, the frequency of antigen‐specific T cells was calculated by subtracting the mean number of spots in the control wells from the mean number of spots observed in response to antigen. Prior to the vaccination, ≥ 5 antigen‐specific T cells were considered a positive response (+ in Table 2) and ≥ 100 antigen‐specific T cells were considered a highly positive response (++). Upregulation of an immune response to the vaccinations (↑) was defined to be a ≥ 1.5‐fold increase of antigen‐specific spot count and ≥ 5 antigen‐specific T cells after vaccinations. For determination of antigen‐specific T cells by multimer staining, the percentage of CD8^+^ or CD4^+^ T cells stained positive with a control multimer was subtracted from the percentage of cells stained positive with the specific multimer. Prior to the vaccination, ≥ 0.1% antigen‐specific T cells were considered a positive response (+ in Table 2) and ≥ 1% antigen‐specific T cells were considered a highly positive response (++). Upregulation of an immune response to the vaccinations (↑) was defined to be a ≥ 2‐fold increase of multimer‐positive CD8^+^ or CD4^+^ T cells and ≥ 0.1% antigen‐specific T cells after vaccinations. Downregulation of an immune response to the vaccinations (↓) was defined to be a ≥ 2‐fold decrease of multimer‐positive CD8^+^ or CD4^+^ T cells. An immune response to a specific antigen was defined by upregulation of the ELISpot and/or multimer response to the respective antigen (Table 2). In order to compare survival data of this single‐arm trial to that of AML patients with very similar characteristics, a carefully matched cohort of 88 patients from the AML‐CG registry was selected according to the following criteria: CR/CRi/CRp after intensive induction therapy; no allo‐HSCT in CR1; duration of remission at least as long as in the trial population; non‐favorable risk type; ECOG 0 or 1; and age at diagnosis 18–75. A comparison of patient characteristics between the DC study cohort and the AML‐CG registry cohort is depicted in Supplementary table 3. Differences in survival between different groups were tested by log‐rank test.

## Conflict of Interest

DJS is employed by Medigene Immunotherapies GmbH, and holds patents and receives royalties for DC vaccines. All other authors declare that they have no conflict of interest.

## Authors' contributions

FSL, WH, DJS, GK, IB and MS designed the clinical trial. FSL, TA, VLB, TK, GW, BW, GK, IB and MS performed the clinical trial. FMS, FSL, MR, KD, CA, NPK, KS, AM, SB, MBo, MBr, MHMH and MS acquired and analysed the data. FSL, FMS, MR, KD and DG performed the statistical analysis. FSL, FMS, MR, KD, and MS designed the figures. FSL, FMS, MR and MS wrote the manuscript.

## Ethics approval and consent to participate

The study was approved by the ethics committee of the LMU Munich and by the Paul‐Ehrlich‐Institute in Langen, Germany. 

## Consent for publication

All patients provided written informed consent.

## Availability of data and material

The data sets used and/or analysed during the current study are available from the corresponding author on reasonable request.
