# Supplementary material for: Toll‐like receptor 7/8‐matured RNA‐transduced dendritic cells as post‐remission therapy in acute myeloid leukaemia: results of a phase I trial
Source: Clin Transl Immunology. 2020 Mar 3;9(3):e1117. doi: 10.1002/cti2.1117 (PMC7053229; doi:10.1002/cti2.1117)
Supplement: Supplementary file 1 [file CTI2-9-e1117-s001.pdf]

Supplementary Materials:

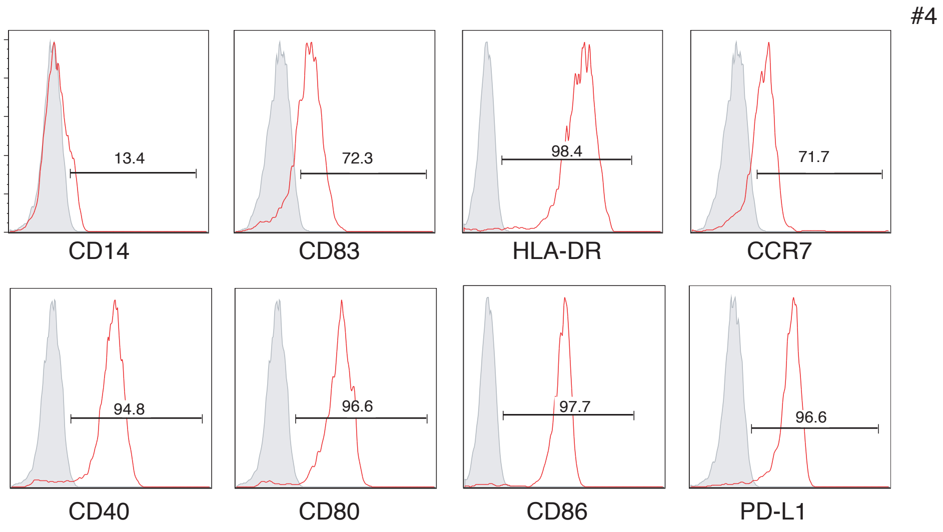

Supplementary figure 1. DC phenotype: Expression of surface molecules detected on DCs by flow cytometry in a representative patient sample.

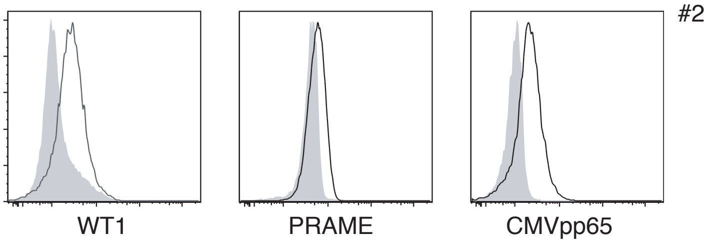

Supplementary figure 2. DC antigen expression: Freshly thawed DCs were intracellularly stained for the proteins translated from the electroporated RNA. DCs electroporated with one of the other two RNA species (PRAME as control for WT-1, CMV as control for PRAME, and WT1 as control for CMVpp65) served as control. A representative patient sample is shown.

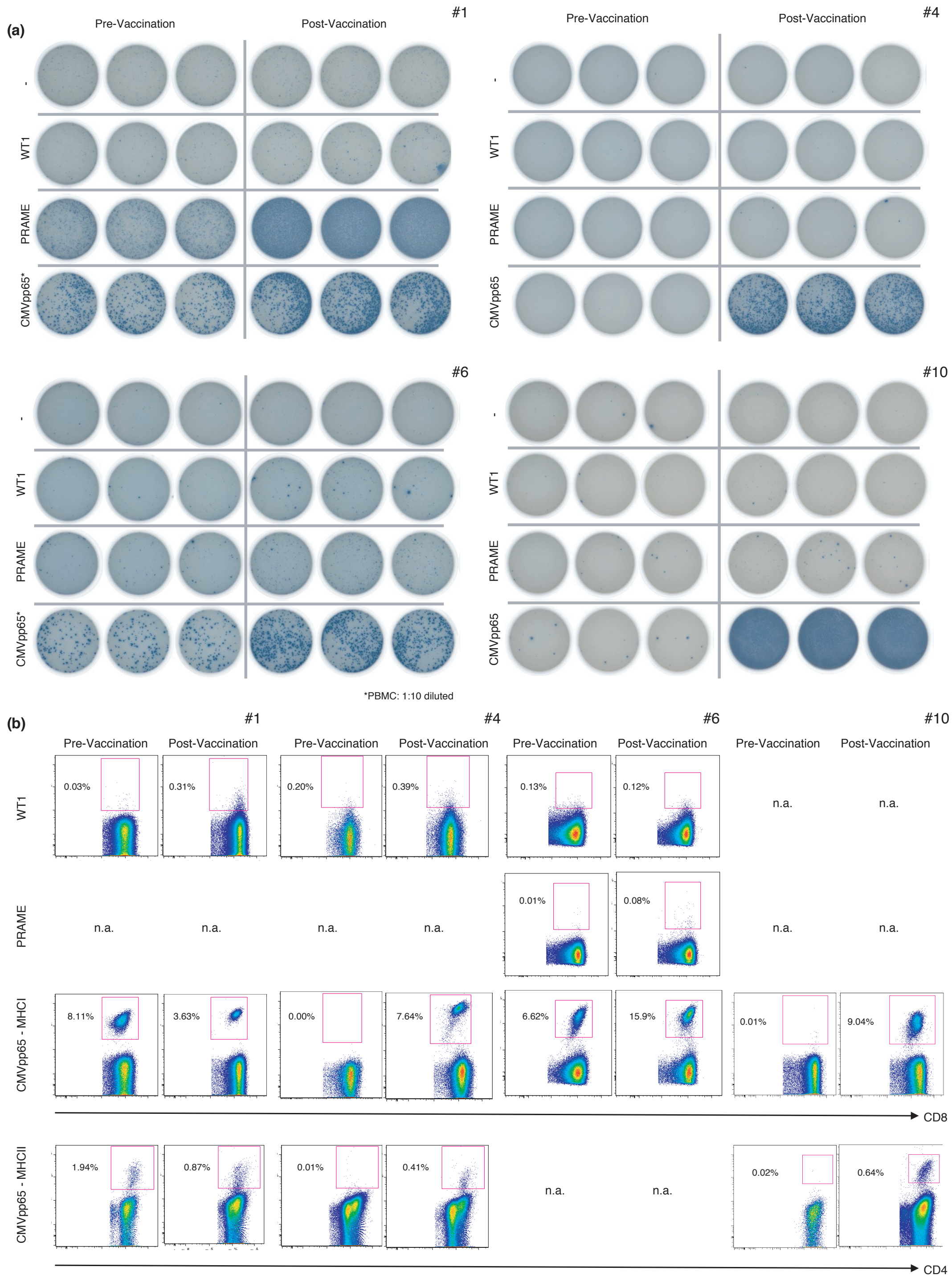

Supplementary figure 3. Immunmonitoring data for patients #1, #4, #6, and #10. PBMCs isolated before and after vaccination were tested for antigen-specific T cells **(a)** by Elispot and **(b)** by multimer staining.

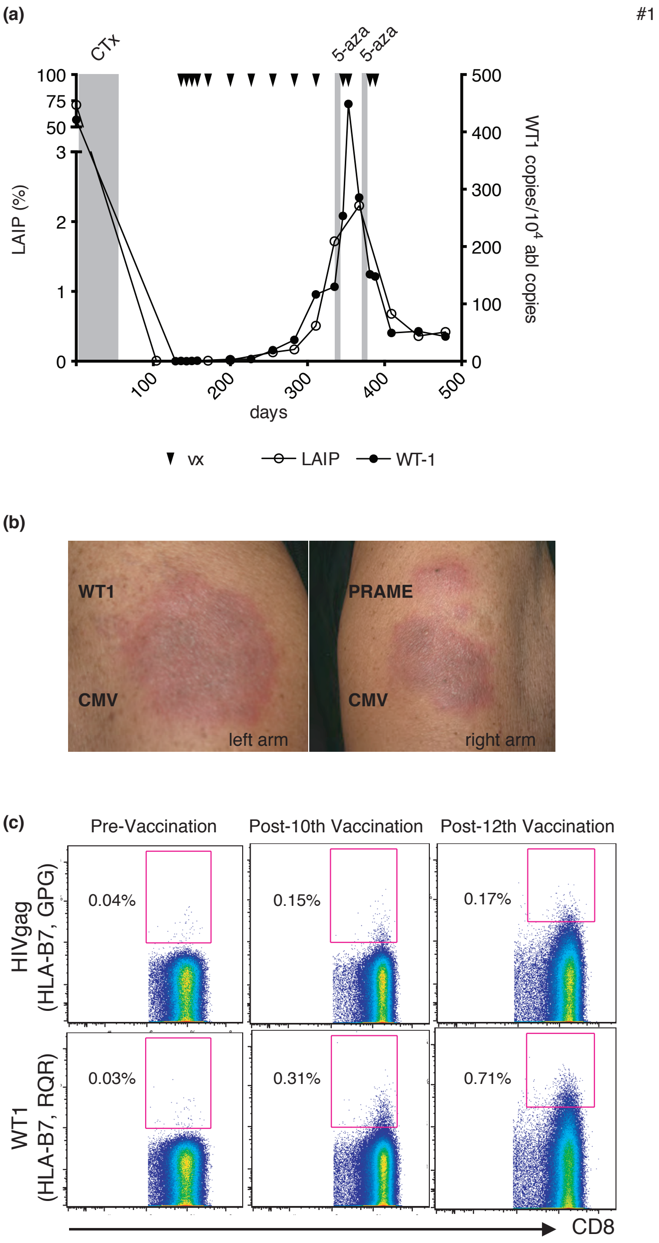

Supplementary figure 4. Individual treatment attempt with combination of 5-azacytidine and DC vaccination in patient #1. (a) Course of LAIP and WT1 MRD, showing MRD relapse after 10 vaccinations and MRD conversion after the combination therapy. **(b)** Enhanced local reaction to the vaccination after preceding 5-azacytidine therapy. **(c)** Increase in WT1-specific T cells after the combination therapy as measured by multimer.

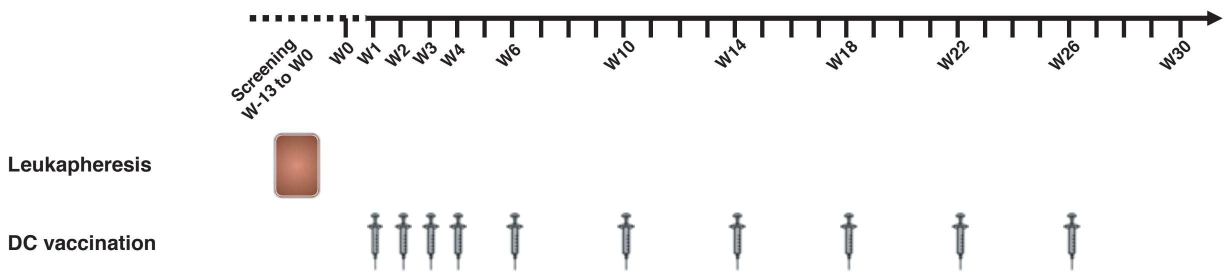

Supplementary figure 5. Vaccination schedule

	Leukocyte count (GxL^-1)	Monocytes (%)	WBC (x10^10)	Monocyte yield (x10^9)	DC recovery after electroporation (x10^8)	Potential vaccinations	Leukapheresis to Vx1 (d)	CR to Vx1 (d)
#1	7.6	11	1.4	3.4	3.76	14	24	82
#2	5.9	7	1	2.2	1.27	6	25	104
#3	2.3	7	0.8	2.7	3.96	14	n.a.	n.a.
#4	3.7	11	1	3.4	5.45	22	32	149
#5	n.a.	n.a.	n.a.	n.a.	n.a.	n.a.	n.a.	n.a.
#6	10.5	4	1.7	5.1	5.68	25	24	176
#7	5.9	13	1.5	3.6	1.85	10	18	34
#8	9.4	6	2.7	7.5	2.86	10	n.a.	n.a.
#9	4.1	10	1.1	3.6	3.39	14	25	97
#10	2.6	11	1.4	3.8	3.54	16	38	96
#11	5.19	7	0.9	3.9	4.84	24	25	116
#12	4.45	7	2.8	6.6	5.22	24	26	205
#13	3.26	9	0.6	1.0	2.38	11	19	185

Supplementary table 1. Leukapheresis data. Leukocyte count and Monocytes refer to the patient's peripheral blood values at the screening visit before leukapheresis. WBC = White blood cells and Monocyte yield refer to the leukapheresis product. Potential vaccinations = Number of sets of three batches each for vaccinations with DCs loaded with all three antigens. Vx1, First vaccination.

Adverse Event	Grade	n (% of n=10)
Vaccine site reaction (erythema, induration, pruritus)	1	10 (100)
Musculoskeletal pain	1–2	6 (60)
Skin reactions (erythema, pruritus) outside of vaccine sites	1	5 (50)
Diarrhea	1–2	4 (40)
Fatigue	1	4 (40)
Headache	1–2	3 (30)
Vertigo	1–2	3 (30)
Arthralgia	1	3 (30)
Cough	1	3 (30)
Nausea and vomiting	1	3 (30)
Respiratory infection	1	3 (30)
Night sweats	1	2 (20)
Pyrexia	1, 3	2 (20)

Supplementary table 2. Adverse events reported during the study. Listed are all adverse events that were observed in ≥ 2 patients and were at least possibly related to the investigational medicinal product.

	DC study cohort	AML-CG cohort
**No. of patients**	13	88
**Age, years**		
Median (range)	62 (44-79)	62 (25-75)
**Patients age, N (%)**		
≤ 65 years	9 (69)	52 (59)
> 65 years	4 (31)	36 (41)
**Sex Female/male, N (%)**	4/9 (31/69)	40/48 (45/55)
**ECOG, N (%)**		
0	2 (15)	24 (27)
1	10 (77)	64 (73)
2	1 (8)	0 (0)
**ELN, N (%)**		
Favorable	2 (15)	0 (0)
IntermediateI	7 (54)	42 (48)
intermediateII	3 (23)	30 (34)
Adverse	1 (8)	16 (18)
**Molecular aberrations, N (%):**		
**NPM1 mutation**		
Pos.	1 (8)	28 (35)
Neg.	11 (92)	53 (65)
Missing/unknown	1	7
**FLT3-ITD**		
Pos.	3 (33)	23 (28)
Neg.	6 (67)	58 (72)
Missing/unknown	4	7
**FAB, N (%)**		
M0	2 (15)	7 (9)
M1	5 (38)	18 (22)
M2	2 (15)	23 (28)
M3	0 (0)	0 (0)
M4	1 (8)	13 (16)
M5	0 (0)	10 (12)
M6	0 (0)	0 (0)
M7	0 (0)	1 (1)
s-AML (MDS)	3 (23)	9 (11)
Missing/unknown	0	7
**Leukocytes at dx (GxL^-1)** Median (range)	3 (1-94)	8 (1-292)
**LDH, UxL^-1** Median (range)	232 (181-2401)	388 (63-8078)

Supplementary table 3. Patient characteristics of the trial cohort in comparison with the matched cohort from the AML-CG registry.

	A	B	C	DRB1	DQB1	DPB1
**#1**	24:02, 30:01	**07:02**, 13:02	06:02, 07:02	**07:01**, 15:01	02:02, 06:02	04:01, 04:02
**#2**	**02:01**, 03:01	44:05, 56:01	01:02, 02:02	**07:01**, 16:01	05:02, 03:03	04:01, 10:01
**#3**	02:01, 24:02	14:01, 40:01	03:04, 08:02	07:01	02:02, 03:03	04:01
**#4**	03:01, 23:01	**07:02**, 44:03	04:01, 07:02	**07:01**, 15:01	02:02, 06:02	04:01
**#5**	02:01, 03:01	07:02	07:02	15:01	06:02	02:01, 04:01
**#6**	**02:01**	15:01, 18:01	03:04, 07:01	04:01, 14:54	03:02, 05:03	04:01
**#7**	03:01, 25:01	13:02, 18:01	06:02, 12:03	**07:01**, 15:01	02:02, 06:02	03:01, 04:01
**#8**	01:01, 03:01	08:01, 40:01	07:01, 15:02	08:01, 15:01	04:02, 06:02	04:01, 04:02
**#9**	03:01, 26:08	15:01, 40:01	03:04, 04:01	01:01, 11:03	03:01, 05:01	03:01, 04:02
**#10**	**01:01**	49:01, 57:01	06:02, 07:22	**07:01,** 13:02	03:03, 06:04	02:01, 04:01
**#11**	**02:01**, 32:01	44:02, 51:01	05:01, 15:02	**07:01**, 11:01	03:01, 03:03	03:01, 04:01
**#12**	**01:01**, 31:01	27:05, 39:01	01:02, 12:03	07:01, **13:01**	02:02, 06:03	04:01
**#13**	11:01, **24:02**	**07:02**, 27:05	02:02, 07:02	13:01, 15:01	06:02, 06:03	03:01, 04:02

Supplementary table 4. HLA-typing of the patients. Multimer staining was performed against the HLAs highlighted in bold with corresponding multimers.

Parameter	Method	Specification
**Total cell count in 500 μL**	Cell Dyn Ruby (Abott)	> 2x10^6
**Viability**	Tryphan blue (Ph. Eur.)	> 60%
**CD80 positive**	Flow Cytometry (BD)	> 60%
**Microbiological contamination**	Bactec System (BD)	Negative
**Contaminating cells (NK, T and B cells)**	Flow Cytometry (BD)	< 20%
**Mycoplasma testing**	16SrDNA PCR and sequencing	Negative

Supplementary table 5. Release Criteria for the DC vaccine.

	Reagent	Manufacturer	City, Country
**Antibodies**	anti-CD14 (FITC, 61D3)	eBioscience	San Diego, CA, USA
	anti-CD40 (PE, clone 5C3)	eBioscience	San Diego, CA, USA
	anti-CD80 (PE, L307.4)	BD Biosciences	Heidelberg, Germany
	anti-CD83 (APC, HB15)	BD Biosciences	Heidelberg, Germany
	anti-CD86 (PB, IT2.2)	BioLegend	San Diego, CA, USA
	anti-CD274 (FITC, MIH1)	BD Biosciences	Heidelberg, Germany
	anti-CCR7 (APC, FR11-11E8)	Miltenyi Biotec	Bergisch Gladbach, Germany
	anti-HLA-DR (PE, LN3)	BioLegend	San Diego, CA, USA
	anti-CD3 (APC, UCHT1)	BioLegend	San Diego, CA, USA
	anti-CD4 (FITC, VIT4)	Miltenyi Biotec	Bergisch Gladbach, Germany
	anti-CD8 (PerCP-eFluor710, SK1)	eBioscience	San Diego, CA, USA
	Live/Dead Aqua	Invitrogen	Carlsbad, CA, USA
	FcR Blocking Reagent	Miltenyi Biotec	Bergisch Gladbach, Germany
	anti-HCMV ppUL83	Biomerieux	Marcy-l'Étoile, France
	anti-WT1 (6F-H2)	Agilent	Santa Clara, CA, USA
	anti-PRAME (ab89097)	Abcam	Cambridge, UK
	AF647-conjugated anti-mouse F(ab)2	Dianova	Hamburg, Germany
	CMVpp65 (A*01:01-YSEHPTFTSQY)	ProImmune	Oxford, UK
	CMVpp65 (A*02:01-NLVPMVATV)	Immudex	Copenhagen, Denmark
	CMVpp65 (A*24:02-QYDPVAALF)	Immudex	Copenhagen, Denmark
	CMVpp65 (B*07:02- TPRVTGGGAM)	Immudex	Copenhagen, Denmark
	CMVpp65 (DRB1*07:01-EPDVYYTSAFVFPTK)	NIH Tetramer Facility	Atlanta, GA, USA
	WT1 (A*02:01-RMFPNAPYL)	Immudex	Copenhagen, Denmark
	WT1 (A*02:01-VLDFAPPGA)	Immudex	Copenhagen, Denmark
	WT1 (A*24:02-CYTWNQMNL)	Immudex	Copenhagen, Denmark
	WT1 (B*07:02- RQRPHPGAL)	Immudex	Copenhagen, Denmark
	PRAME (A*02:01-VLDGLDVLL)	Immudex	Copenhagen, Denmark
	PRAME (A*02:01-ALYVDSLFFL)	Immudex	Copenhagen, Denmark
	HIV-Gag (A*02:01-SLYNTVATL)	Immudex	Copenhagen, Denmark
	HIV-Gag (A*24:02-RYLKDQQLL)	Immudex	Copenhagen, Denmark
	HIV-Gag (B*07:02-GPGHKARVL)	Immudex	Copenhagen, Denmark
	CLIP (DRB1*07:01-PVSKMRMATPLLMQA)	NIH Tetramer Facility	Atlanta, GA, USA
**Primer**	Roche FastStart Essential DNA Probes Master (# 06402682001)	Roche Diagnostics	Basel, Switzerland
	Real time ready singe Assays - Roche PRAME Assay ID: 117436, config. # 100104279	Roche Diagnostics	Basel, Switzerland
	Real time ready singe Assays - Roche Abl1 Assay ID: 144473, config. # 100104288	Roche Diagnostics	Basel, Switzerland
	WT1 forward primer 5´-cgctattcgcaatcagggtta-3´	MetaBion International AG	Martinsried, Germany
	WT1 reverse primer 5´-gggcgtgtgaccgtagct-3´	MetaBion International AG	Martinsried, Germany
	WT1 probe 5´-FAM-agcacggtcaccttcgacgg-BHQ-1-3´	MetaBion International AG	Martinsried, Germany
	cABL Taq forward primer 5´-cct ttt cgt tgc act gta tga ttt-3´	MetaBion International AG	Martinsried, Germany
	cABL Taq reverse primer 5´-cgcc taa gac ccg gag ctt tt-3´	MetaBion International AG	Martinsried, Germany
	ABL1 probe 5´-FAM-tgg cca gtg gag ata aca ctc taa gca taa cta aag g-BHQ-1-3´	MetaBion International AG	Martinsried, Germany

Supplementary table 6. List of antibodies and primers used in the study.
